# The effect of paired associative stimulation with a high-intensity cortical component and a high-frequency peripheral component on heart rate and heart rate variability in healthy subjects

**DOI:** 10.3389/fresc.2023.1200958

**Published:** 2023-07-26

**Authors:** P. Haakana, K. Holopainen, A. Nätkynmäki, E. Kirveskari, M. P. Tarvainen, A. Shulga

**Affiliations:** ^1^ BioMag Laboratory, HUS Diagnostic Center, Helsinki University Hospital, University of Helsinki and Aalto University School of Science, Helsinki, Finland; ^2^ Motion Analysis Laboratory, New Children’s Hospital, Helsinki University Hospital and University of Helsinki, Helsinki, Finland; ^3^ HUS Medical Imaging Center, Clinical Neurophysiology, Clinical Neurosciences, Helsinki University Hospital and University of Helsinki, Helsinki, Finland; ^4^ Department of Technical Physics, University of Eastern Finland, Kuopio, Finland; ^5^ Department of Clinical Physiology and Nuclear Medicine, Kuopio University Hospital, Kuopio, Finland; ^6^ Department of Physical and Rehabilitation Medicine, Helsinki University Hospital and University of Helsinki, Helsinki, Finland

**Keywords:** transcranial magnetic stimulation, peripheral nerve stimulation, parasympathetic activity, autonomic nervous system, cardiovascular function

## Abstract

**Objective:**

A novel protocol for paired associative stimulation (PAS), called high PAS, consists of high-intensity transcranial magnetic stimulation (TMS) and high-frequency peripheral nerve stimulation (PNS). High PAS was developed for spinal cord injury rehabilitation and targets plastic changes in stimulated pathways in the corticospinal tract, which improves motor function. As therapy interventions can last many weeks, it is important to fully understand the effects of high PAS, including its effect on the cardiovascular system. Heart rate variability (HRV) has been used to measure changes in both sympathetic and parasympathetic systems.

**Methods:**

We used short-term HRV measurements to evaluate the effects of one 20-min session of high PAS on 17 healthy individuals. HRV was recorded for 5 min before (PRE), during (STIM), immediately after (POST), 30 min after (POST30), and 60 min after (POST60) the stimulation. Five participants repeated the HRV setup with sham stimulation.

**Results:**

A significant decrease in low-frequency (LF) power (n.u.) (*p* = 0.002), low-frequency to high-frequency (HF) ratio (*p* = 0.017), in Poincaré plot [the standard deviation of RR intervals perpendicular to (SD1) and along (SD2) the line of identity SD2/SD1 ratio *p* < 0.001], and an increase in HF power (n.u.) (*p* = 0.002) were observed between PRE and STIM conditions; these changes were fully reversible immediately after stimulation. PRE to POST by 3% (*p* = 0.015) and continued to decline until POST60 by 5% (*p* = 0.011). LF power (ms^2^) (*p* = 0.017) and SD2 (*p* = 0.015) decreased from PRE to STIM and increased from PRE to POST (*p* = 0.025 and *p* = 0.017, respectively). The results from sham PAS exhibited a trend similar to active high-PAS stimulation.

**Conclusions:**

High PAS does not have sustained effects during 60-min follow-up on cardiovascular functions, as measured by HRV. None of the short-term results indicates activation of the sympathetic nervous system in healthy individuals. Observed changes in HRV indicate higher parasympathetic activity during stimulation, which is reversible, and is plausibly explained by the fact that the participants spend 20 min without moving, talking, or using phones while being stimulated.

## 1. Introduction

Paired associative stimulation (PAS) is a non-invasive stimulation method (1) that may have potential for rehabilitation of patients with neurological disorders such as spinal cord injury (SCI) (2–7) and stroke (8, 9). PAS has also been used to investigate the mechanisms of disease and plasticity in Parkinson's disease (10), dystonia (11), and Huntington's disease (12), among others. Conventional PAS combines simultaneous transcranial magnetic stimulation (TMS) over the motor cortex (M1) and peripheral nerve stimulation (PNS) (1). Stimulation pairs are timed to synchronously activate presynaptic and postsynaptic neurons, leading to improved synaptic efficacy and inducing plastic changes in the stimulated pathways such as the corticospinal tract (5, 12, 13). The effects of PAS on the corticospinal tract can be evaluated as changes in motor-evoked potentials (MEPs) (14), which are the responses to motor-cortex stimulation recorded from the corresponding muscles (15).

Our group has developed a novel PAS protocol, called high PAS, which consists of a high-intensity TMS (100% of maximum stimulator output, MSO) and a high-frequency (100 Hz) PNS (5, 16, 17). We developed this protocol for SCI rehabilitation. The protocol is usually applied to a patient for many weeks (2, 4, 5, 18, 19), which highlights the importance of safety issues of the applied stimulation method, especially when the used TMS intensity is considered. Previously, no studies have reported the effects of PAS on cardiac functions or heart rate variability (HRV), which is the fluctuation in time between heartbeats. Single-pulse TMS is safe (20), as no effects on blood pressure and heart rate have been reported (21). However, no studies have reported the effects of single-pulse TMS on HRV (22). Studies that combine TMS with functional magnetic resonance imaging (fMRI) have revealed that a 1-Hz suprathreshold TMS on the primary motor cortex (M1) creates blood-oxygenation-level-dependent (BOLD) activation mainly in the ipsilateral hemisphere (23). Suprathreshold 4-Hz repetitive TMS also showed an increase of BOLD activation in M1 and in ipsilateral corticocortical connections (24).

HRV can be assessed in the time domain and frequency domain and using nonlinear Poincaré plots. In the time domain, the normal-to-normal (NN) intervals between successive ECG QRS complexes are analyzed. Standard deviation of NN intervals (SDNN) reflects the overall variability. In the root mean square of successive differences between RR intervals (RMSSD), the number of intervals greater than 50 ms (NN50) and the proportion of NN50 with the total number of NN intervals (pNN50) reflect beat-to-beat variability (25). An increase in beat-to-beat time-domain variables indicates activation of the parasympathetic nervous system. In certain circumstances, the SDNN may also increase because of the activation of the sympathetic nervous system. In frequency-domain analysis, HRV data are divided into the following three spectral components: very low frequency (VLF, 0–0.04 Hz), LF (0.04–0.15 Hz), and HF (0.15–0.4 Hz) (25). Breathing frequency usually falls under the HF band. Different frequency bands can be assessed as the relationship between sympathetic and vagally controlled LF and vagally controlled HF, calculated as an LF/HF relation or as normalized values expressing LF or HF contribution to total power excluding the VLF component (25). Oscillations in the HF band are driven by the vagus nerve and follow respiratory frequency. The LF component represents mostly sympathetic modulation (26). Therefore, assuming that respiratory frequency is within the HF band, the LF/HF ratio (usually between 1 and 2) and the normalized LF power represent sympathovagal balance (27). The ratio between the Poincaré plot standard deviations SD2 and SD1 (SD2/SD1) is an alternative approach for assessing sympathovagal balance, but without the assumption on respiratory rate (28).

HRV has been used to measure the functions of the autonomic nervous system, which consists of the sympathetic nervous system (responsible for increase in blood pressure and increase in heart rate) and parasympathetic nervous system (responsible for relaxation and slowing heart rate) (29). Cardiac function is controlled by various areas of the central nervous system, such as the anterior insula, hypothalamus, periaqueductal gray matter, parabrachial nucleus, and several regions of the medulla (30). Heart rate is controlled by both sympathetic and parasympathetic nerves at the sinoatrial node. The vagus nerve serves as an inhibitory control, decreasing the heart rate (31). The preganglionic parasympathetic cardiovascular neurons are located in the nucleus ambiguous, and their axons reach through the superior cervical and inferior thoracic rami to the cardiac ganglia (30). Parasympathetic influence inhibits the heart rate, as shown in studies where sympathetic inputs are blocked (25), and leads to a lower heart rate, thus conserving energy at rest (32). Parasympathetic activation is mediated by a synaptic release of acetylcholine with a short latency, whereas sympathetic activation results in a synaptic release of noradrenaline and an increase in heart rate (33).

Decreased HRV is associated with several diseases and all-cause mortality. Lower HRV can be an indicator of insufficient adaptability in the autonomic nervous system (32, 33). This decrease has been observed in variables in both time domain (SDNN) and frequency domain (HF, absolute power) parameters (25). However, simple activities such as talking or reading can affect HRV by altering breathing frequency (34). Normal HRV in healthy individuals is considered to be adaptable and self-regulatory (35), to improve emotion regulation (36), and to be flexible with environmental changes (37). For short-term measurements, a spectral analysis is used as an estimate for autonomic influence on HRV (38). In spectral analysis, HRV can be divided into VLF, LF, and HF components.

The purpose of this study was to identify acute cardiovascular effects, specifically HRV, of one 20-min session of high PAS on healthy participants. We developed a high PAS consisting of a high-intensity TMS and a high-frequency peripheral component for SCI rehabilitation. As stimulation therapies may last for several weeks, it is important to identify any possible unfavorable effects of high PAS on autonomic cardiac functions. The aim of this study was to validate the protocol on healthy individuals before investigating the effects on an SCI population.

## 2. Materials and methods

### 2.1. Participants

Seventeen healthy subjects (10 females, seven males, mean age 29 ± 7.8 years) were recruited for this study. Self-reported handedness (15 right, two left) and self-reported footedness (answer to the question “with which leg do you prefer to kick a ball?”; 15 right, two left) were documented. Exclusion criteria were brain pathology, implanted devices, regular medication, neurological diseases, cardiac diseases, psychiatric diseases, drug abuse, and pregnancy. Each participant provided a written informed consent prior to participation. The Medical Ethics Committee of the Helsinki University Hospital approved the study.

### 2.2. Experimental setup

**Figure 1:**
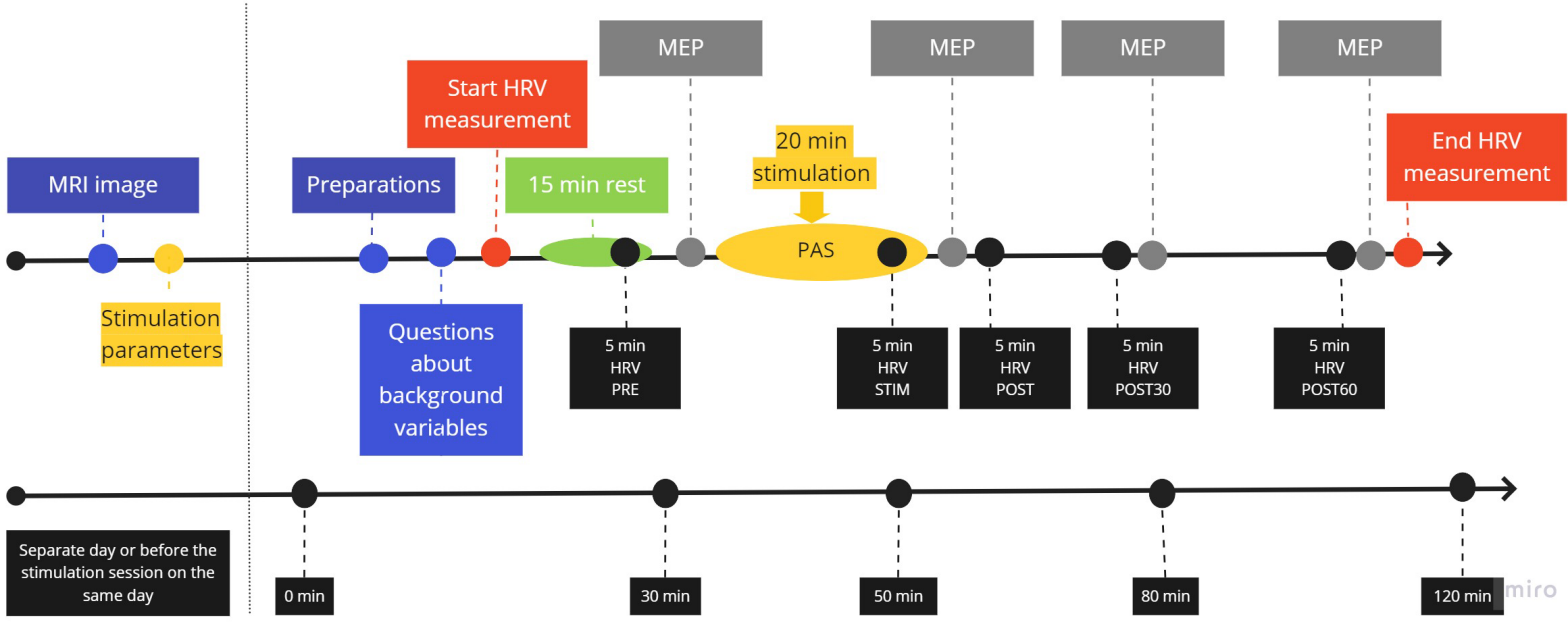
Protocol flowchart. An MRI image was taken and stimulation parameters were determined on a separate day or during the stimulation session before protocol initiation. Preparations included seating the participant comfortably, setting up HRV, attaching stimulation and EMG electrodes, and revising TMS stimulation location in the navigation system.

The experimental session lasted up to 120 min, including 20 min of active stimulation (Figure 1). Prior to the stimulation, the participants were seated for 15 min to ensure that reliable data were generated from the heart rate and HRV without the effects of commute, climbing stairs, or other physical activities. Electrodes for electrocardiogram (ECG) monitor (Bittium Faros 180, Oulu, Finland) and electromyogram (EMG) for HRV and MEP recordings, respectively, were attached. We asked questions about variables that might possibly affect HRV at the beginning of the session (Table 1) and, from the answers to these questions, we confirmed that no confounding factors would cause a change in HRV variables. Five participants drank 1–4 cups of coffee before coming to the laboratory; these participants were habitual coffee drinkers.

**Table 1:** List of questions asked in the beginning of each session about variables that might affect HRV measurement.

Questions asked at the beginning of each session
Regularly used medication, if any
Any medication taken during the past 2 days
Have you had pain during the past 24 h;
if yes, where, and how intense (scale 1–10)?
Stress level experienced
Are the previous 24 h representative of normal daily physical activity?
How many hours slept last night?
Does the day of the measurement represent your ordinary day?
Have you had any caffeinated drinks today?

Responses are presented in Supplementary Table 1.

#### 2.2.1. Transcranial magnetic stimulation

Navigated TMS (eXimia magnetic stimulator, Nexstim Ltd., Helsinki, Finland) was delivered using single pulses at 100% of the maximum stimulator output (MSO), corresponding to 172 V/m ± 2 measured 25 mm below the coil (NextStim NBS manual). Stimulation was performed with a figure-of-eight coil with an outer diameter of 70 mm. Individual stimulation parameters were determined on a different day or on the same day before initiation of the session. First, the stimulation target area for the left abductor hallucis muscle representation area was set using the MRI image and the TMS navigation system. The individual resting motor threshold (RMT) was then determined. MEP recordings were performed at 120% of RMT. MEP amplitude was recorded before, immediately after, and at 30 and 60 min after the stimulation.

#### 2.2.2. Peripheral nerve stimulation

PNS was delivered as trains of six pulses (1-ms biphasic square pulses at 100 Hz). Stimulation (Dantec Keypoint® Natus Medical Incorporated, California, USA) was applied to the tibial nerve, which is behind the left medial malleolus. Stimulation intensity was individually determined during the same session with TMS parameters: the lowest intensity where F-responses were detectable was used for stimulation (39). Local anesthesia using 5% lidocaine/prilocaine (EMLA) ointment was offered to the participants to reduce the effect of the sensation of the pricking skin associated with PNS stimulation (40). Sixteen participants used this ointment.

#### 2.2.3. Paired associative stimulation

We applied high PAS as previously described (5, 41). Presentation® software (Neurobehavioral Systems Inc., Albany, USA) was used to sync TMS and PNS pulses to be delivered with individual interstimulus interval (ISI) (41) once every 5 s. Altogether, 240 PAS sequences were administered during the 20-min stimulation. For five participants, HRV was also recorded during sham PAS, where TMS was delivered at 100% MSO with a 5-cm block between the scull and the coil. PNS electrodes did not deliver current.

#### 2.2.4. Heart rate variability

HRV was measured by using a Bittium Faros 180 (Bittium corporation, Oulu, Finland) device using three ECG electrodes (Blue sensor, Ambu A/S, Ballenrup, Denmark) and a sampling frequency of 1,000 Hz. The ECG electrodes and the recording device were attached at the beginning of the session, recording was done throughout the session, and the device was removed at the end of the session. The following 5-min periods were used for HRV analysis: at the end of the 15-min rest period prior to stimulation (PRE), at the end of the 20-min stimulation period (STIM), immediately after post-stimulation MEP measurement (POST), and immediately before post 30 (POST30) and post 60 (POST60) MEP measurement (Figure 1). A representative 5-min segment from the area of interest without any major noise from movement or MEP stimulation was selected for the HRV analysis after obtaining agreement from two investigators. Thus, the timing of the selected analysis segments might vary by minutes within participants in relation to the protocol. HRV data were analyzed by using Kubios HRV Premium software (Kubios Ltd., Kuopio, Finland). On average, the software used beats correction on 1.2% of the data (maximum 2.8%). The analyzed variables are presented in Table 2.

**Table 2:** Description of heart rate parameters, adopted time-domain, frequency domain, and non-linear HRV parameters and their main association to autonomic nervous system function.

HRV parameter	(units)	Description	Association with ANS function
Heart rate parameters			
Mean RR	(ms)	Mean of the selected beat-to-beat RR interval series	PNS↑ and SNS↓
Mean HR	(bpm)	Mean heart rate, inversely proportional to Mean RR	SNS↑ and PNS↓
Max HR	(bpm)	Maximum heart rate (evaluated as 5-beat average)	SNS↑ and PNS↓
Min HR	(bpm)	Minimum heart rate (evaluated as 5-beat average)	SNS↑ and PNS↓
Time-domain HRV parameters			
SDNN	(ms)	Standard deviation of all normal RR intervals (normal-to-normal intervals, NN), demonstrating overall variability	PNS↑ and SNS↕
RMSSD	(ms)	Root mean square of successive differences between RR intervals, demonstrating beat-to-beat variation	PNS↑
NN50	(beats)	Number of consecutive NN interval pairs differing by more than 50 ms	
pNN50	(%)	NN50 divided by the total number of all NN intervals, demonstrating beat-to-beat variation	PNS↑
Frequency-domain HRV parameters			
LF power	(ms^2^)	LF power (frequency range 0.04–0.15 Hz) extracted from RR interval time series power spectrum	SNS↕
LF power	(n.u.)	LF power in normalized units (n.u.) representing the relative power in proportion to total power (TP) minus VLF power: LF [n.u.] = LF power [ms^2^]/(TP [ms^2^]—VLF power [ms^2^])	SNS vs. PNS
HF power	(ms^2^)	High-frequency power (frequency range 0.15–0.4 Hz) (synchronous with respiration); estimates parasympathetic/vagal activation	PNS
HF power	(n.u.)	HF power in normalized units (n.u.) representing the relative power in proportion to TP minus VLF power:HF [n.u.] = HF power [ms^2^]/(TP [ms^2^]—VLF power [ms^2^])	PNS vs. SNS
LF/HF		LF/HF power ratio	SNS vs. PNS
Resp	(Hz)	Respiratory rate estimated from ECG and HRV data	
Nonlinear HRV parameters			
Poincaré SD1	(ms)	In Poincaré plot, the standard deviation of RR intervals	PNS↑
Poincaré SD2	(ms)	Perpendicular to (SD1, demonstrating beat-to-beat variability) and along (SD2, demonstrating overall variability) the line of identity	PNS↑ and SNS↕
SD2/SD1		SD2/SD1 ratio	SNS vs. PNS

SNS, sympathetic nervous system; PNS, parasympathetic nervous system; ↑, indicates ANS activation tends to increase the HRV parameter; ↓, indicates ANS activation tends to decrease the HRV parameter; ↕, diverse association with ANS function, indicates ANS activation may increase or decrease the HRV parameter.

HRV and MEP values were recorded during the 60-min follow-up period at 30 min and at 60 min post stimulation. The participants were asked to sit still, but were allowed to talk, to move a little, and use their phones.

### 2.3. Statistical analysis

Statistical analysis was performed by using IBM SPSS 27 software. Normality of the data was reviewed by using the Kolmogorov–Smirnov test. For normally distributed data, Levene's test showed that homogeneity was not met and thus the Friedman test and Wilcoxon signed-rank test were used with Bonferroni correction. The effect size was reported as Kendall's W for Friedman test and % change from PRE for the Wilcoxon signed-rank test. We compared the change in HRV variables and MEP amplitudes between PRE and STIM, POST, POST30, and POST60 time points. For determining the sex effect, the Mann–Whitney U test was used and *η*^2^ was reported as the effect size. This test was also used for pairwise comparison between PAS and PAS sham sessions.

## 3. Results

HRV frequency limits were set to LF (0.04–0.15 Hz) and HF (0.15–0.40 Hz). For one participant who had higher respiration frequency, we exceptionally set the higher limit to 0.50 Hz. The data recorded from POST30 min were removed for one participant because his respiration frequency fell under the low-frequency band. One participant had an unpleasant sensation and cold sweat during PAS, but the participant did not want the experiment to be stopped. No other adverse events were observed. The significance level was adjusted with Bonferroni correction to 0.012.

The results obtained from 5-min analysis windows for HRV and MEP measurements from different time points are presented in Table 3. Consistent with our previous work (16, 19, 42), PAS induced significant MEP potentiation (*p* < 0.001; Kendall's *W* 0.361). POST, POST30, and POST60 were significantly different from PRE (*p* < 0.001, *p* = 0.025, and *p* = 0.015), respectively. There were no significant differences in mean RR, mean HR, RMSSD, NN50, pNN50, HF power (ms^2^), resp (Hz), and SD1. Friedman's test showed a significant difference for SDNN (*p* = 0.003, Kendall's *W* = 0.234), LF power ms^2^ (*p* < 0.001, Kendall's *W* = 0.379), LF power n.u. (*p* < 0.001, Kendall's *W* = 0.305), HF power n.u. (*p* < 0.001, Kendall's *W* = 0.291), LF/HF ratio (*p* < 0.001, Kendall's *W* = 0.305), SD2 (*p* < 0.001, Kendall's *W* = 0.284), and SD2/SD1 ratio (*p* < 0.001, Kendall's *W* = 0.388). A further analysis with the Wilcoxon signed-rank test showed significant differences between PRE and STIM on HR max (*p* = 0.001), LF power (ms^2^) (*p* = 0.017), LF power n.u. (*p* = 0.002), HF power n.u. (*p* = 0.002), and the SD2/SD1 ratio (*p* = 0.000) (Table 3). HR min continued to decrease POST (*p* = 0.015), POST30 (*p* = 0.028), and POST60 (*p* = 0.011).

**Table 3:** Averages and standard error of mean for selected variables.

Variable *p*-value for Friedman's test	PRE	SEM	STIM	SEM	*p*-Value % change from PRE	POST	SEM	*p*-value % change from PRE	POST30	SEM	*p*-value % change from PRE	POST60	SEM	*p*-value % change from PRE
Stimulation responses														
MEP (mV) <0.001**	628.40	1.90	–	–	–	931.50	2.30	<0.001** (+48.2%)	730.20	2.00	0.025	717.50	2.00	0.015
Heart rate parameters														
Mean RR (ms) 0.082	886.6	26.6	916.0	29.1		886.4	21.7		918.6	26.0		921.8	26.3	
Mean HR (bpm) 0.082	68.7	2.0	66.6	2.1		68.3	1.7		66.2	1.9		66.0	1.9	
Min HR (bpm) 0.005*	60.2	1.7	60.2	1.7	0.868	58.4	1.3	0.015	57.7	1.5	0.028	57.2	1.7	0.011* (−4.7%)
Max HR (bpm) 0.014	82.6	2.7	75.8	2.8	0.001** (−8.3%)	83.4	2.0	0.795	81.7	2.3	0.554	80.1	2.7	0.124
Time-domain HRV parameters														
SDNN (ms) 0.003**	47.9	3.7	40.7	3.8	0.084	54.3	3.9	0.017	49.8	3.5	0.492	51.3	4.3	0.102
RMSSD (ms) 0.279	43.1	4.3	45.0	5.3		45.5	4.7		46.9	3.4		47.2	5.6	
NN50 (beats) 0.253	72.2	11.1	84.1	15.1		77.4	12.8		88.4	10.9		76.8	13.3	
pNN50 (%) 0.306	22.0	3.7	26.2	5.1		23.3	4.0		27.6	3.8		24.3	4.4	
Frequency-domain HRV parameters														
LFpow (ms^2^) <0.001**	1,329.9	202.7	849.6	265.7	0.017	1,936.9	310.7	0.025*	1,451.4	345.7	0.906	1,719.3	305.4	0.093
HFpow (ms^2^) 0.140	709.9	155.3	906.7	172.5		803.8	212.3		839.2	126.3		908.1	224.2	
LFpow (n.u.) <0.001**	65.1	3.9	45.5	4.3	0.002** (−28.4%)	71.0	4.0	0.124	60.0	4.5	0.177	66.0	4.3	0.603
HFpow (n.u.) <0.001**	34.9	3.9	54.3	4.3	0.002** (101.8%)	29.0	4.0	0.124	40.0	4.5	0.177	33.9	4.3	0.653
LF/HF ratio <0.001**	2.8	0.7	1.3	0.4	0.017*	3.8	0.7	0.149	2.4	0.6	0.163	2.8	0.5	0.523
RESP (Hz) 0.893	0.3	0.0	0.3	0.0		0.3	0.0		0.3	0.0		0.3	0.0	
Non-linear HRV parameters														
SD1 (ms) 0.279	30.5	3.1	31.9	3.8		32.2	3.3		33.2	2.4		33.4	4.0	
SD2 (ms) <0.001**	60.0	4.6	46.9	4.5	0.015	69.3	4.7	0.017	61.6	4.7	0.795	63.8	5.0	0.124
SD2/SD1 ratio <0.001**	2.1	0.2	1.6	0.1	<0.000** (−22.9%)	2.3	0.1	0.103	1.9	0.1	0.042	2.1	0.1	0.795

*p*-Values are presented as % change from PRE value, calculated as average of individual % change values. MEP, motor evoked potentials; mV, millivolts; RR, RR interval; the time between two R waves in QRS signal; ms, millisecond; SDNN, standard deviation of NN interval; time normalized RR interval; HR, heart rate, bpm, beats per minutes; RMSSD, root mean square of successive differences; NN50, number of successive RR interval pairs that differ by more than 50 ms; pNN50, NN50 divided by the total number of RR intervals; LF, low frequency; HF, high frequency; n.u., normalized units; RESP, respiration frequency; Hz, hertz; SD1, standard deviation perpendicular to the line of identity (Poincaré plot); SD2, standard deviation along the line of identity (Poincaré plot).

* Statistical difference compared to pre *p* < 0.010, **p < 0.005.

**Figure 2:**
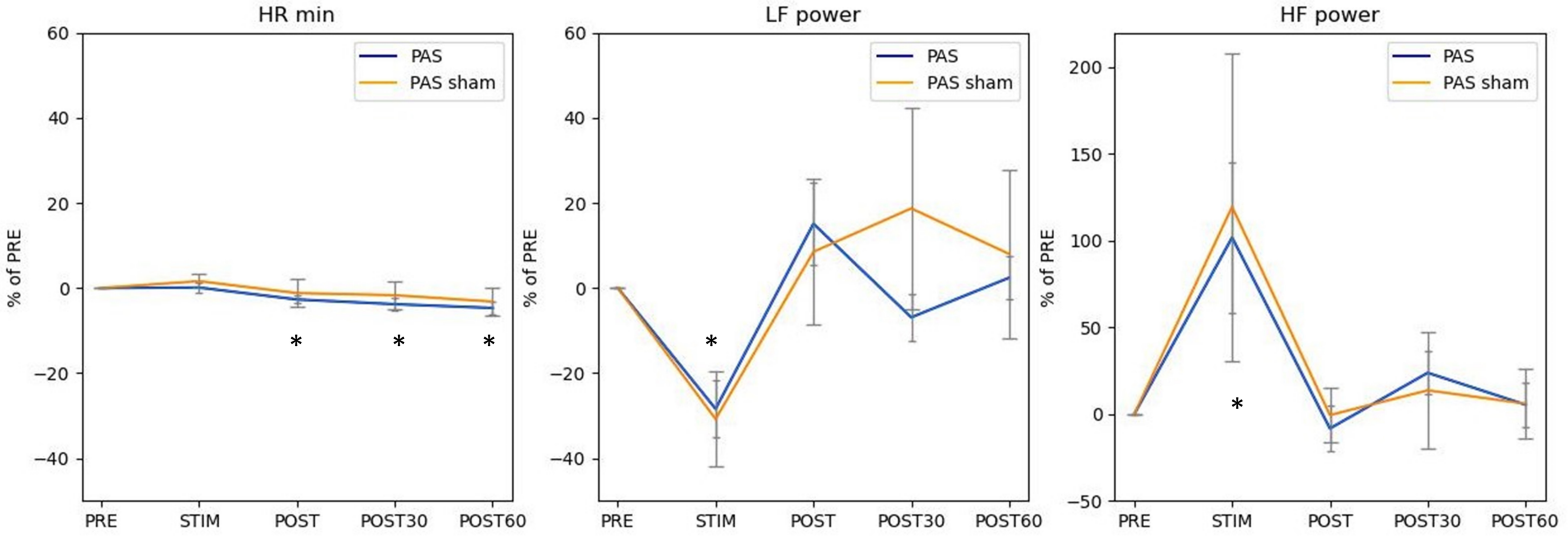
Similar pattern of HR min, LF power (n.u.), and HF power (n.u.) across conditions in both sham (wider error bar caps, *n* = 5), and active stimulation (*n* =17). Results are presented as % of PRE condition with standard error of mean. * Statistical difference compared to pre *p* < 0.05.

The most pronounced but reversible differences were observed between PRE and STIM on LF power n.u. (*p* = 0.002) and HF power n.u. (*p* = 0.002) (Table 3). To assess whether these differences resulted from sitting still and not specifically from PAS, we performed additional sham stimulation in five participants (Figure 2). Although a similar trend was observed for sham stimulation as was the case for active stimulation, because of the smaller number of participants, the changes within the sham session were not significant (Min HR (*p* = 0.277), LF power n.u. (*p* = 0.145), and HF power n.u. (*p* = 0.145). Pairwise comparison with the PAS session was also not significant.

The effect of sex on HRV variables is presented in Table 4. Females had an overall higher HR (*p* < 0.001), a smaller LF power (*p* < 0.001), and a correspondingly higher HF power (*p* < 0.001). Females also had a higher respiration frequency (*p* = 0.007). In the time-domain variables, there were no significant differences between male and female participants. There was a significant difference between males and females at PRE stimulation for LF power (n.u.) (*p* = 0.013), HF power (n.u.) (*p* = 0.013), and LF/HF ratio (*p* = 0.013).

**Table 4:** The effect of sex on different variables from all data points.

Variable	Sex	Average ± SEM	*p*-Value	Effect size *η*^2^
Heart rate parameters				
Mean RR (ms)	Male	970 ± 17	<0.001***	0.170
	Female	878 ± 14		
Mean HR (bpm)	Male	62.5 ± 1.2	<0.001***	0.170
	Female	69.2 ± 1.0		
Min HR (bpm)	Male	54.5 ± 0.7	<0.001***	0.193
	Female	60.7 ± 1.0		
Max HR (bpm)	Male	75.6 ± 1.8	0.002**	0.123
	Female	82.9 ± 1.4		
Time-domain HRV parameters				
SDNN (ms)	Male	50.9 ± 3.1	0.415	
	Female	48.4 ± 2.3		
RMSSD (ms)	Male	43.9 ± 3.4	0.376	
	Female	48.0 ± 2.8		
NN50 (beats)	Male	70.0 ± 9.2	0.144	
	Female	89.4 ± 7.4		
pNN50 (%)	Male	22.9 ± 3.2	0.247	
	Female	27.1 ± 2.4		
Frequency-domain HRV parameters				
LFpow (ms^2^)	Male	1,837 ± 268	0.038*	0.055
	Female	1,268 ± 152		
HFpow (ms^2^)	Male	657 ± 103	0.033*	0.057
	Female	983 ± 116		
LFpow (n.u.)	Male	70.8 ± 3.0	<0.001**	0.159
	Female	54.8 ± 2.6		
HFpow (n.u.)	Male	29.1 ± 3.0	<0.001**	0.160
	Female	45.1 ± 2.6		
LF/HF ratio	Male	3.8 ± 0.5	0.001**	0.160
	Female	1.9 ± 0.3		
RESP (Hz)	Male	0.26 ± 0.01	0.010*	0.84
	Female	0.29 ± 0.01		
Nonlinear HRV parameters				
SD1 (ms)	Male	31.1 ± 2.4	0.379	
	Female	34.0 ± 2.0		
SD2 (ms)	Male	64.2 ± 4.1	0.225	
	Female	58.8 ± 2.8		
SD2/SD1 ratio	Male	2.2 ± 0.1	0.012*	0.080
	Female	1.8 ± 0.1		

*N* = 16, six males and 10 females.

* Statistical difference compared with PRE *p* < 0.010, **p < 0.005.

## 4. Discussion

Our results show that any effect of PAS on HRV is short term, and that the parasympathetic system is transiently activated during the PAS session. Although most changes appeared in the HRV frequency domain, the time domain and nonlinear parameters showed significant changes during and after stimulation compared with PRE. Most of the changes reverted to prestimulation levels immediately after stimulation. HR min continued to decline throughout the observation period up to post 60 min, although the extent of decline was only 5%. PAS did not cause any activation of the sympathetic system.

Our results are similar to that of Yoshida et al. (22). After stimulation, they found that HF power increased after a 25 min supine, indicating activation of the parasympathetic nervous system. They delivered TMS with 0.2 Hz at 90% of the motor threshold, whereas the intensity was maximum stimulator output in our study (22). Other neuromodulation methods, such as anodal transcranial direct current (tDCS), have been shown to reduce the heart rate and modify HRV with an increase in SDNN (43, 44), although there is no consensus in the literature (45). In the frequency domain, an increase in the LF, LF/HF ratio (43), and HF (44) has been observed with anodal tDCS.

Activation of the parasympathetic system may be attributed to 20 min of sitting still and listening to the cyclical sound of TMS. In our previous clinical work, some patients had a tendency to fall asleep during high-PAS treatment (5). The participants were asked to sit still after stimulation, although minimal but little movement (such as changing position, talking, and using a phone) was permitted. Bernardi et al. studied the effects of silent reading, free talking, and controlled breathing on HRV and reported a decrease in RR and an increase in the LF band with silent reading and an increase in both during free talking (34); the effect appeared to be mediated by changes in respiratory frequency. In this study, no changes were observed in respiratory frequency between measurement time points.

Sham control with five participants showed a similar pattern in results, when compared with a regular high-PAS session; a similar significance level as in active treatment was not achieved because of the lower participant number. This indicates that PAS stimulation alone does not plausibly create profound changes in the cardiovascular system. Probable mechanisms for HRV changes that could have activated the parasympathetic nervous system are remaining still for 20 min without talking, using a phone, or moving while listening to the cyclic sound from the stimulator.

There are several confounding factors in HRV measurements that can influence the results. Slow breathing (around six cycles per minute) can increase vagally mediated HRV variables such as RMSSD and LF (46). In our study, no change was observed in breathing frequency between time points. Age, sex, and physical or mental illness may influence HR and HRV (45). Females have a higher heart rate and lower RR interval variability, and in the frequency domain, females have a lower total power (47). In this study, significant differences between male and female participants were found in heart rate parameters and in frequency-domain HRV variables. HRV was not compared between individuals in this study, but changes in HRV were observed over time intraindividually. Five of 17 participants did not remember the instructions to not drink coffee on the morning of the measurement. However, the influence of caffeine on autonomic control of the heart is inconclusive in the literature (48). For example, Rauh et al. have shown that caffeine does not alter HRV in habitual drinkers (49).

High PAS has been developed as a rehabilitation tool for patients with neurological conditions, especially spinal cord injury. In this study, we investigated the effects of high PAS on acute cardiovascular effects in healthy participants. However, more research is needed to determine the relevance of these results in different patient groups.

## 5. Conclusion

This study revealed that high PAS is safe and does not show any cardiovascular effects over a 60-min follow-up. An increase in the HF band indicates activation of the parasympathetic system during the stimulation, plausibly due to sitting still without any disturbance. The results indicated that HRV parameters return to normal as soon as normal activity is resumed after PAS stimulation. This also indicates that high PAS is not excessively stressful for the participants, which is important and beneficial for its further development as a therapy for neurological patients.

## Data Availability

The raw data supporting the conclusions of this article will be made available by the authors without any undue reservation.
